# Bedarf und Vorhersagbarkeit von Magnetresonanztomographieuntersuchungen bei Patienten mit implantiertem Neurostimulator

**DOI:** 10.1007/s00482-021-00598-7

**Published:** 2021-11-03

**Authors:** Marco Reining, Dirk Winkler, Joachim Böttcher, Jürgen Meixensberger, Michael Kretzschmar

**Affiliations:** 1 Klinik für Schmerz- und Palliativmedizin, SRH Wald-Klinikum Gera GmbH, Straße des Friedens 122, 07549 Gera, Deutschland; 2 Klinik und Poliklinik für Neurochirurgie, Universitätsklinikum Leipzig, Leipzig, Deutschland; 3 Friedrich-Schiller-Universität Jena, Jena, Deutschland; 4 Campus Gera, SRH Hochschule für Gesundheit, Gera, Deutschland

**Keywords:** Elektrische Rückenmarkstimulation, Diagnostische Bildgebung, Neuromodulation, Versorgungsforschung, Schmerztherapie, Spinal cord stimulation, Diagnostic imaging, Neuromodulation, Health services research, Pain medicine

## Abstract

**Hintergrund:**

Bei steigender Zahl von Magnetresonanztomographie(MRT)-Untersuchungen in der deutschen Gesamtbevölkerung gibt es keine Daten zum Untersuchungsbedarf von Patienten mit implantiertem Neurostimulator in Deutschland. Publizierte Daten aus den USA legen einen hohen Bedarf nahe. Die eingeschränkte MRT-Zulassung der Implantate ist in der täglichen Praxis ein häufiges Problem.

**Ziel:**

Im Fokus steht der MRT-Bedarf dieser Schmerzpatienten und die Vorhersagbarkeit zum Zeitpunkt der Implantation.

**Material und Methoden:**

Es erfolgte eine retrospektive Auswertung der Datenbank unseres Klinikinformationssystems. Gesucht wurden alle im Zeitraum November 2011 bis März 2019 in unserem Klinikum angeforderten MRT-Untersuchungen für Patienten mit implantiertem Neurostimulator. Zudem erfolgte ein Abgleich mit den im gleichen Zeitraum durchgeführten Implantationen derartiger Stimulationssysteme.

**Ergebnisse:**

Es konnten 171 durchgeführte MRT-Untersuchungen und 22 Anforderungen ohne nachfolgende Untersuchung ausgewertet werden. Bei 83 von 294 Patienten, die in unserem Zentrum mit implantierten Neurostimulatoren versorgt wurden, erfolgte mindestens eine MRT-Untersuchung in unserem Klinikum. Wir beobachten eine stetig steigende Nachfrage. In 111 von 171 durchgeführten Untersuchungen (65 %) bestand kein Zusammenhang zwischen der zur Implantation führenden Indikation und der Indikation für die MRT. Eine Vorhersagbarkeit konnte nur bei 43 von 193 MRT-Anforderungen (22 %) unterstellt werden.

**Diskussion:**

Patienten mit implantiertem Neurostimulator haben auch in Deutschland einen hohen Bedarf an MRT-Diagnostik, welcher zum Zeitpunkt der Implantation nicht vorhersagbar ist. Daher sollten ausschließlich MRT-taugliche Systeme implantiert werden. Die Industrie ist aufgefordert, die Implantate und deren Zulassungen an den Bedarf anzupassen.

## Abstract (en)

**Background:**

With an increasing number of magnetic resonance imaging (MRI) examinations in the general population, there are no data available regarding the requirements of patients with implanted neurostimulators in Germany. Published data from the United States of America suggest a high need. The limited approval for MRI scans of implants are a common problem.

**Objective:**

The focus is on the MRI needs of these pain patients and the predictability at the time of implantation.

**Material and method:**

We carried out a retrospective evaluation of the database of our hospital information system. We searched for all MRI requests for patients with an implanted neurostimulator between November 2011 and March 2019. In addition, we compared these data with the implantation of neurostimulators in the same period.

**Results:**

We identified 171 MRI examinations and 22 requests without a subsequent examination. Out of 294 (28%) patients implanted in our center 83 had at least 1 MRI scan in our hospital. We observed a steadily increasing demand. In 111 of 171 (65%) performed examinations, there was no association between the indications leading to neurostimulator implantation and to MRI. A predictability could only be assumed for 43 of 193 (22%) MRI requests.

**Conclusion:**

In Germany, patients with an implanted neurostimulator have a high need for MRI diagnostics which cannot be predicted at the time of implantation. Therefore, only MRI-conditional systems should be implanted. The manufacturers need to adapt the implants and their approval to requirements.

## Hintergrund und Fragestellung

Die Neurostimulation ist ein etabliertes Verfahren zur Behandlung neuropathischer und ischämischer Schmerzen. Ein häufiges klinisches Problem in der Nachsorge ist die fehlende oder eingeschränkte Zulassung dieser Implantate für Magnetresonanztomographie(MRT)-Untersuchungen. Amerikanische Registerdaten legen einen hohen MRT-Bedarf dieser Patienten nahe, Daten aus Deutschland wurden bisher nicht publiziert. Auch die Frage nach der Vorhersagbarkeit zukünftiger MRT-Scans zum Zeitpunkt der Implantation ist bisher nicht beantwortet, dies könnte aber eine Rolle bei der Wahl der Implantate spielen.

Die Magnetresonanztomographie hat sich aufgrund des exzellenten Weichteilkontrasts bei vielen Erkrankungen zum diagnostischen Verfahren der ersten Wahl entwickelt [1]. Die Bedeutung der MRT belegen auch stetig steigende Untersuchungszahlen weltweit, in Deutschland stieg die Zahl der MRT-Untersuchungen pro 1000 Einwohner von 97,0 im Jahr 2009 auf geschätzt 145,1 im Jahr 2018 [15].

Gleichzeitig sind die elektrische Rückenmarkstimulation („spinal cord stimulation“ [SCS]), die Hinterwurzelganglionstimulation („dorsal root ganglion stimulation“ [DRG-S]), die periphere Nervenstimulation (PNS) und die periphere Nervenfeldstimulation (PNFS) etablierte invasive Verfahren zur Therapie chronischer Schmerzen [5, 8, 11]. Im Jahr 2019 wurden in Deutschland 1954 Neurostimulatoren zur rückenmarksnahen Stimulation (SCS und DRG-S) implantiert [21]. Daten zur Prävalenz in der deutschen Gesamtbevölkerung sind nicht verfügbar, wir schätzen, dass derzeit mindestens 10.000 Patienten mit einem Neurostimulator versorgt sind. Aktuelle deutsche Leitlinien zur elektrischen Rückenmarkstimulation empfehlen eine Behandlung insbesondere bei persistierenden Schmerzen nach Wirbelsäuleneingriffen („failed back surgery syndrome“ [FBSS]), komplexem regionalem Schmerzsyndrom (CRPS) Typ I und II, ischämischen Schmerzen bei Angina pectoris und peripherer arterieller Verschlusserkrankung (pAVK) und sonstigen neuropathischen Schmerzen [7, 8].

Die meisten derzeit verfügbaren Generatoren haben eine sehr limitierte Zulassung für MRT-Untersuchungen mit vielen Einschränkungen („MR conditional“), älteren Modellen fehlt häufig die MRT-Zulassung. Hinsichtlich des Zulassungsstatus muss immer das Gesamtsystem mit allen Komponenten betrachtet werden: ein zugelassener Generator und eine zugelassene Elektrode bedeuten nicht automatisch eine Zulassung des Gesamtsystems. Die Einschränkungen können z. B. die Art und Lage der Elektroden, die zu untersuchenden Körperregionen, spezifische Anforderungen an das MRT-System (z. B. geschlossene horizontale Systeme, Magnetfeldstärke) und nutzbare Spulen betreffen. Zusätzlich gelten Grenzen für die technischen Parameter (z. B. spezifische Absorptionsrate, Gradientenfelder, Untersuchungsdauer). Auskunft hierzu geben die Bedienungsanleitungen oder einschlägige Übersichtsarbeiten [18, 19]. Aufgrund dieser vielfältigen Einschränkungen sind nur wenige MRT-Untersuchungen innerhalb der Zulassung möglich [17].

Bisher gibt es nur zwei Arbeiten, welche sich auf Basis einer kommerziellen US-amerikanischen Datenbank (Truven Reuters MarketScan® Database, Truven Health Analytics, Ann Arbor, MI, USA) mit dem Bedarf an MRT-Untersuchungen für diese Patientengruppe beschäftigen: Desai und Kollegen schätzen, dass 82–84 % der mit einem Neurostimulator versorgten Patienten innerhalb der ersten fünf Jahre mindestens eine MRT-Untersuchung benötigen [6]. Farber und Kollegen resümieren, dass Patienten mit FBSS, welches die häufigste Indikation zur Neurostimulation ist, einen im Vergleich mit der Durchschnittsbevölkerung 1,73-fachen MRT-Bedarf haben. Patienten mit einem Neurostimulator erhalten aber nur halb so viele MRT-Untersuchungen wie Patienten ohne Neurostimulator [9].

Diese Arbeit soll zwei Fragen beantworten:

- Wie hoch ist der MRT-Bedarf von Patienten mit implantiertem Neurostimulator in Deutschland?
- Ist es möglich, den zukünftigen MRT-Bedarf zum Zeitpunkt der Implantation des Neurostimulators vorherzusagen?

## Materialien und Methoden

### Datenakquise

Nach positivem Ethikvotum erfolgte eine Suche in der Datenbank unseres Klinikinformationssystems nach MRT-Anforderungen und gleichzeitig bestehenden Hinweisen auf das Vorhandensein eines Neurostimulators (Schlüsselwörter, Diagnose- bzw. Prozedurenschlüssel, Kontakt mit unserer Abteilung) für den Zeitraum von November 2011 (Gründung unseres Schmerzzentrums) bis März 2019. Bei allen Suchergebnissen überprüften wir die gesamte Patientenakte und extrahierten daraus die benötigten Daten.

Zusätzlich erfolgte eine weitere Suche in der Datenbank für den gleichen Zeitraum nach allen Operationen im Zusammenhang mit einem Neurostimulator über die Prozedurenschlüssel (5-039.*). Hier wurde die Patientenakte des jeweiligen Aufenthalts überprüft und die notwendigen Daten extrahiert.

Alle Zuordnungen zu Gruppen erfolgten durch den Erstautor und wurden durch den Letztautor überprüft, Fälle mit differenter Einschätzung wurden im gesamten Autorenteam diskutiert und die Zuordnung festgelegt.

### Statistik

Zur Datenerfassung und -analyse verwendeten wir eine handelsübliche Tabellenkalkulationssoftware mit allen aktuellen Updates (Microsoft Excel® 365, 64 Bit; Microsoft, Inc., Redmond, WA, USA).

Es erfolgte eine Beratung durch einen Statistiker, dieser empfahl eine rein deskriptive Darstellung unserer Daten.

## Ergebnisse

### Identifikation und Charakterisierung der Studienteilnehmer

Die Suche nach MRT-Anforderungen in der Datenbank unseres Klinikinformationssystems lieferte 1024 Treffer, in 193 Fällen konnte das Vorhandensein eines implantierten Neurostimulators bestätigt werden. In 22 Fällen erfolgte aus verschiedenen Gründen keine MRT-Untersuchung, sodass 171 durchgeführte MRT-Untersuchungen und 22 Anforderungen ohne resultierende MRT-Untersuchung ausgewertet werden konnten.

Die 171 MRT-Untersuchungen betrafen 100 verschiedene Patienten (49 Männer, 51 Frauen) im Alter von 28 bis 87 Jahren (Median: 57 Jahre). Häufigste Indikationen für die Implantation des Neurostimulators waren ein FBSS (*n* = 49), Wirbelsäulenbeschwerden ohne vorangegangene Operation (*n* = 14), CRPS (*n* = 9) und periphere Nervenschäden (*n* = 9).

Die Suche nach den durchgeführten Operationen im Zusammenhang mit Neurostimulatoren ergab 1065 Treffer, hiervon betreffen 294 Eingriffe die Erstimplantation eines Neurostimulators.

### Charakterisierung des MRT-Bedarfs

**Figure 1:**
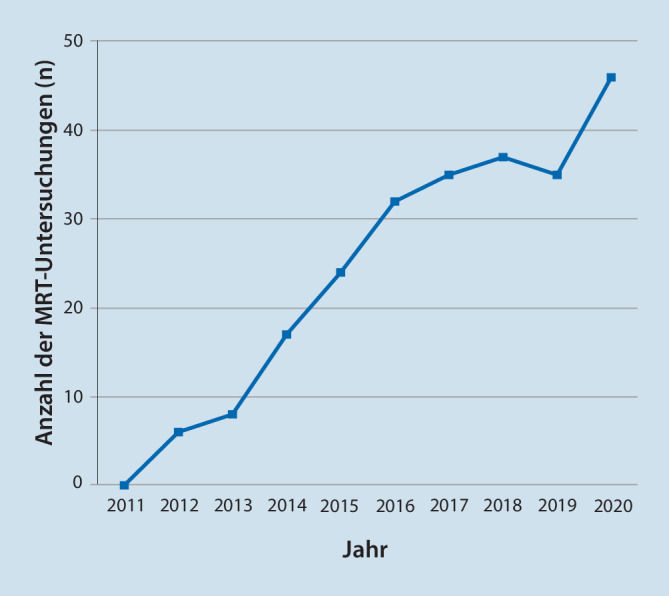

**Figure 2:**
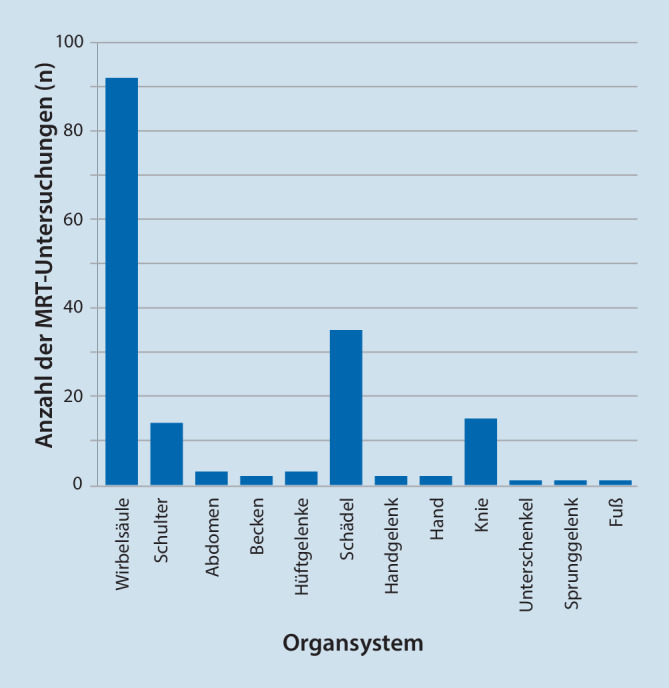

Wir beobachten in unserer Einrichtung eine stetig zunehmende Zahl von MRT-Untersuchungen bei Patienten mit implantiertem Neurostimulator (vgl. Abb. 1). Die untersuchten Körperregionen sind in Abb. 2 dargestellt. 121 der 171 durchgeführten MRT-Untersuchungen (70,8 %) können dem Fachgebiet Orthopädie/Unfallchirurgie einschließlich Wirbelsäulenchirurgie zugeordnet werden, weitere 37 Untersuchungen (21,6 %) sind dem Fachgebiet Neurologie zuzuordnen. 8 Fälle (4,7 %) betrafen onkologische Fragestellungen, weitere Anforderungen stammen aus den Fachgebieten Hals-Nasen-Ohren-Heilkunde (*n* = 2), Endokrinologie (*n* = 2) und Psychiatrie (*n* = 1).

Wie bereits oben beschrieben, wurde die Erstimplantation eines Neurostimulators bei 294 Patienten in unserem Schmerzzentrum vorgenommen. 84 dieser 294 Patienten (28,6 %) benötigten mindestens eine MRT-Untersuchung, insgesamt wurden 141 MRT-Untersuchungen an in unserem Klinikum implantierten Patienten durchgeführt. Die übrigen 30 MRT-Untersuchungen verteilen sich auf 16 extern implantierte Patienten, welche bei uns wegen Schließung der implantierenden Einrichtung (*n* = 20), Wechsel des Wohnorts (*n* = 1) oder Neueröffnung einer näher am Wohnort gelegenen Einrichtung (*n* = 2) weiterbetreut werden. Hinzu kommen Patienten, welche im implantierenden externen Zentrum betreut werden, aber zum Zeitpunkt der MRT-Untersuchung in unserem Klinikum stationär behandelt wurden (*n* = 7).

Wir haben beispielhaft die Untersuchungszahlen für das Kalenderjahr 2018 (letztes vollständiges Kalenderjahr im Beobachtungszeitraum) näher analysiert: Es wurden 37 MRT-Untersuchungen an 29 verschiedenen Patienten durchgeführt, davon wurden 3 Patienten mit 5 MRT-Untersuchungen nicht in unserem Haus implantiert. Seit der Gründung unseres Zentrums im Jahr 2011 bis zum Jahresende 2018 haben wir insgesamt 288 Patienten einen Neurostimulator erstmals implantiert. Im Jahr 2018 erhielten somit 9,0 % der bei uns mit einem Neurostimulator versorgten Patienten mindestens eine MRT-Untersuchung.

In 93 von 171 Fällen (54,4 %) konnten für die weitere Behandlung entscheidende Befunde erhoben werden. Neben neuen, wegweisenden Diagnosen zählten hierzu auch Negativbefunde, zum Beispiel in der Tumornachsorge oder beim Ausschluss einer klinisch gut begründeten Verdachtsdiagnose. Ein konkretes Beispiel für die zuletzt genannte Konstellation ist der Ausschluss einer Spondylodiszitis bei Rückenschmerzen, Fieber und massiv erhöhten Entzündungswerten im Labor. In 72 Fällen (42,1 %) konnten keine therapieverändernden Befunde erhoben werden, und in 6 Fällen (3,5 %) war aufgrund frühzeitiger Untersuchungsabbrüche keine Befundung möglich.

**Figure 3:**
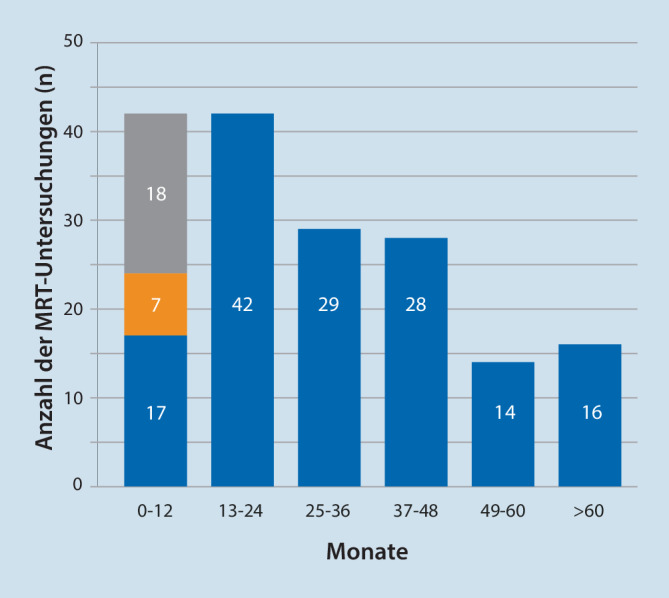

Die Zeit zwischen Implantation des Neurostimulators und der MRT-Untersuchung ist in Abb. 3 dargestellt, der Median beträgt 25 Monate (Spannweite: 0–119 Monate). In 4 Fällen war die MRT-Untersuchung bereits nach Implantation der Elektrode, aber noch vor Implantation des Impulsgenerators erforderlich; diese Fälle haben wir mit 0 Monaten berücksichtigt, bei Ausschluss dieser Fälle ergibt sich ein Median von 27 Monaten bei gleicher Spannweite. In einem Fall ist das genaue Datum der Implantation bei extern erfolgtem Eingriff nicht mehr nachvollziehbar; dieser Patient gehört eindeutig in die Gruppe > 60 Monate und hat daher keinen Einfluss auf die Qualität unserer Daten und deren Auswertung.

### Vorhersagbarkeit von MRT-Untersuchungen

Es wurden alle 193 MRT-Anforderungen ausgewertet. Die retrospektive Analyse der Vorhersagbarkeit stützte sich auf 3 Fragen:

- Ein Zusammenhang zwischen der Indikation zur Implantation des Neurostimulators und der Indikation für die MRT-Untersuchung konnte nur in 70 von 193 Fällen (26,3 %) unterstellt werden.
- Die Unterteilung in akute oder chronische Erkrankungen ist in Tab. 1 dargestellt.
- Die zur MRT-Diagnostik führende chronische Erkrankung war in 58 von 106 Fällen (54,7 %) zum Zeitpunkt der Implantation des Neurostimulators bekannt.

**Table 1:** 

–	*n* (%)
Neues akut aufgetretenes gesundheitliches Problem	87 (45,1)
Akute Verschlechterung einer vorbestehenden chronischen Erkrankung	46 (23,8)
Chronische Erkrankung allein	60 (31,1)

Letztendlich können wir nur in 43 Fällen (22,3 %) eine Vorhersehbarkeit der Notwendigkeit einer MRT-Diagnostik unterstellen. Diese betrafen:

- 29 Patienten mit chronischen Rückenbeschwerden: Durch die behandelnden Fachärzte wurde die eindeutige, kaum widerlegbare Indikation zu Kontrolluntersuchungen gestellt.
- 12 Patienten mit vorbestehenden chronischen Erkrankungen mit absehbarem Bedarf an Kontrolluntersuchungen (z. B. onkologische Patienten, zerebrale Gefäßmalformationen).
- Je 1 Patient mit akuter Verletzung und mit akuter Exazerbation einer vorbestehenden chronischen Erkrankung.

## Diskussion

### MRT-Bedarf von Patienten mit implantiertem Neurostimulator

Mit dieser retrospektiven Arbeit publizieren wir erstmals Daten zum MRT-Bedarf von Patienten mit implantiertem Neurostimulator in Deutschland. Wir konnten zeigen, dass ca. 29 % der in unserem Klinikum implantierten Patienten im Betrachtungszeitraum von ca. 7½ Jahren mindestens eine MRT-Untersuchung in unserem Klinikum erhalten haben. Wir haben deutliche Anhaltspunkte, dass unsere Zahlen den MRT-Bedarf dieser Patientengruppe unterschätzen. Zum einen erhalten wir regelmäßig Anfragen aus anderen Kliniken zur MRT-Zulassung unserer Implantate, da dort die Indikation zur MRT-Untersuchung gestellt wurde. Zum anderen berichten uns unsere Patienten immer wieder von extern erfolgten MRT-Untersuchungen.

Im Vergleich mit den publizierten US-amerikanischen Registerdaten [6, 9], nach denen 82–84 % der Patienten mit Neurostimulator eine MRT innerhalb von 5 Jahren benötigen, erscheint der MRT-Bedarf unserer Patienten geringer. In Deutschland erhielten etwa 7,19 % der Bevölkerung im Jahr 2009 mindestens eine MRT-Untersuchung [2]. Von 2009 bis 2018 ist die Zahl der MRT-Untersuchungen pro 1000 Einwohner in Deutschland um ca. 50 % gestiegen [15], somit ist zu erwarten, dass 10–11 % der Bevölkerung mindestens eine MRT-Untersuchung im Jahr 2018 hatten. Auch wir beobachten bei unseren Patienten eine deutliche Zunahme an durchgeführten MRT-Untersuchungen, im Jahr 2018 erhielten 9,0 % unserer Patienten mindestens eine MRT-Untersuchung; dies entspricht nahezu dem MRT-Bedarf der deutschen Durchschnittsbevölkerung, wobei extern erfolgte MRT-Untersuchungen unberücksichtigt bleiben.

Bei der Interpretation unserer Daten muss die stark eingeschränkte Zulassung der Neurostimulationssysteme für MRT-Untersuchungen berücksichtigt werden [19]. 66,7 % der bei uns durchgeführten MRT-Untersuchungen betreffen den Rumpfbereich, welcher in Bezug auf die MRT-Zulassung der Implantate häufig problematisch ist. In vielen Fällen ist die Untersuchung nicht durch die Zulassung gedeckt, sodass zunächst die zugelassenen nichtinvasiven Verfahren der zweiten Wahl (Röntgen, Computertomographie, Sonographie) ausgeschöpft wurden und häufig auf die eigentlich indizierte MRT-Untersuchung aufgrund der fehlenden Zulassung verzichtet wurde. Farber und Kollegen beschreiben einen hohen Bedarf an Bildgebung und einen geringeren Anteil an MRT-Bildgebung [9], diese Beobachtung können wir grundsätzlich bestätigen. Studien zur Sicherheit von MRT-Untersuchungen sind rar und beziehen sich mit unterschiedlicher Schwerpunktsetzung zumeist auf ältere, heute nur noch selten anzutreffende Implantate [4, 13, 14, 17, 22].

Die stark eingeschränkte Zulassung führt zu einem Mehraufwand beim Radiologen: Häufig müssen am MRT-Gerät spezielle Einstellungen der technischen Parameter entsprechend den Empfehlungen der Hersteller vorgenommen werden, welche zuvor aufwendig recherchiert werden müssen. Dies führt dazu, dass diese Untersuchungen vielfach einen deutlich höheren Zeitaufwand erfordern, welcher schlecht mit den häufig eng getakteten Terminplanungen in Einklang zu bringen ist. Zudem bestehen bei vielen Radiologen Bedenken hinsichtlich der Patientensicherheit. Daher ist es nach wie vor nicht ohne Weiteres möglich, diese Patienten einer solchen Diagnostik zuzuführen.

In unserer Kohorte führte die MRT-Untersuchung bei 54,4 % der Fälle zu eindeutig therapierelevanten Befunden. Der Anteil relevanter Befunde variiert stark abhängig vom untersuchten Organsystem, der Fragestellung, Indikationsstellung und der Verfügbarkeit von MRT-Diagnostik. Daher ist ein Vergleich dieses Parameters bei unserem selektierten Patientengut mangels passender Vergleichsgruppen nicht möglich. Zudem kann auch der Ausschluss einer potenziell gefährlichen Erkrankung auf den zweiten Blick therapierelevant sein, sodass dieser Zahlenwert kein geeigneter Qualitätsparameter ist.

Im Vergleich zur Durchschnittsbevölkerung ist der hohe Anteil an Untersuchungen der Wirbelsäule auffällig [2]. Dies ist sicherlich durch die Selektion des Patientenguts zu erklären. Ebenfalls überraschend ist der hohe MRT-Bedarf in den ersten 2,5 Jahren nach der Erstimplantation eines Neurostimulators, hierfür haben wir derzeit noch keine plausible Erklärung.

### Vorhersagbarkeit künftiger MRT-Untersuchungen

Zur Frage der Vorhersagbarkeit zukünftig erforderlicher MRT-Untersuchungen zum Zeitpunkt der Implantation des Neurostimulators gibt es nach kritischer Durchsicht der verfügbaren Literatur noch keine Arbeiten.

Ein Großteil (63,7 %) der erforderlichen MRT-Untersuchungen steht in keinem Zusammenhang mit der (Schmerz‑)Indikation für den Neurostimulator. Zumeist nicht vorhersehbare neue akute Ereignisse (45,1 %) und auch akute Ereignisse bei vorbestehenden chronischen Erkrankungen (23,8 %) sind für einen überwiegenden Teil der Untersuchungen verantwortlich. Die chronischen Erkrankungen waren nur in 54,7 % der Fälle zum Zeitpunkt der Implantation bereits bekannt. Bei zum Zeitpunkt der Implantation noch nicht bekannten Erkrankungen kann die Vorhersagbarkeit ebenfalls eindeutig verneint werden.

Retrospektiv waren nur 22,3 % der MRT-Anforderungen vorhersehbar, eine prospektive Einschätzung des zukünftigen MRT-Bedarfs vor der Implantation erscheint deutlich schwieriger. Daher sollte aus unserer Sicht immer ein Neurostimulator mit möglichst umfassender MRT-Zulassung implantiert werden. Ein besonderer Bedarf liegt in der Bildgebung des Rumpfbereichs. Die Hersteller der Implantate sind aufgefordert, bei künftigen Produktentwicklungen diesen MRT-Bedarf zu berücksichtigen und die Implantate bzw. deren MRT-Zulassung zu verbessern. Wünschenswert ist eine Untersuchung des gesamten Körpers im Normalmodus des MRT-Geräts (spezifische Absorptionsrate ≤ 2 W/kg) unabhängig von der Platzierung der Elektroden. Zukünftig wird aufgrund besserer Bildqualität auch die Zulassung für Untersuchungen in 3‑Tesla-MRT-Geräten eine Rolle spielen [20], für einige Indikationen konnte bereits ein Vorteil der Bildgebung mit höherer Feldstärke gezeigt werden [3, 10, 12, 16].

Wesentliche Limitation unserer Arbeit ist das monozentrische, retrospektive und primär auf die Untersuchung der Sicherheit dieser MRT-Untersuchungen ausgelegte Studiendesign.

## Fazit für die Praxis

- Patienten mit implantiertem Neurostimulator haben einen relevanten MRT-Bedarf.
- Die MRT-Untersuchungen erfolgen häufig kurz nach der Implantation und betreffen überwiegend den Rumpf (inklusive Wirbelsäule).
- Der künftige Bedarf an MRT-Untersuchungen ist zum Zeitpunkt der Implantation in der überwiegenden Zahl der Fälle nicht vorhersehbar.
- Daher sollten ausschließlich MRT-kompatible Neurostimulatoren implantiert werden.
- Die Hersteller sind aufgefordert, die Implantate und deren MRT-Zulassung an die klinischen Bedürfnisse anzupassen und um die Verwendung in MRT-Geräten mit 3‑Tesla-Technik zu erweitern.
